# Generalized Richards model for predicting COVID-19 dynamics in Saudi Arabia based on particle swarm optimization Algorithm

**DOI:** 10.3934/publichealth.2020064

**Published:** 2020-11-02

**Authors:** Rafat Zreiq, Souad Kamel, Sahbi Boubaker, Asma A Al-Shammary, Fahad D Algahtani, Fares Alshammari

**Affiliations:** 1 Department of Public Health, College of Public Health and Health Informatics, University of Ha'il, Ha'il, Saudi Arabia; 2 Molecular Diagnostic and Personalized Therapeutics Unit, University of Ha'il, Ha'il, Saudi Arabia; 3 Department of Computer & Networks Engineering, College of Computer Science and Engineering, University of Jeddah, Jeddah, Saudi Arabia; 4 Laboratory of Control, Electric Systems and Environment, National College of Engineering of Monastir, Tunisia; 5 Department of Biology, Faculty of Science, University of Ha'il, Ha'il, Saudi Arabia; 6 Department of Health Informatics, College of Public Health and Health Informatics, University of Ha'il, Ha'il, Saudi Arabia

**Keywords:** COVID-19 dynamics, prediction, Generalized Richards Model (GRM), projected end date, Particle Swarm Optimization (PSO)

## Abstract

COVID-19 pandemic is spreading around the world becoming thus a serious concern for health, economic and social systems worldwide. In such situation, predicting as accurately as possible the future dynamics of the virus is a challenging problem for scientists and decision-makers. In this paper, four phenomenological epidemic models as well as Suspected-Infected-Recovered (SIR) model are investigated for predicting the cumulative number of infected cases in Saudi Arabia in addition to the probable end-date of the outbreak. The prediction problem is formulated as an optimization framework and solved using a Particle Swarm Optimization (PSO) algorithm. The Generalized Richards Model (GRM) has been found to be the best one in achieving two objectives: first, fitting the collected data (covering 223 days between March 2^nd^ and October 10, 2020) with the lowest mean absolute percentage error (MAPE = 3.2889%), the highest coefficient of determination (R^2^ = 0.9953) and the lowest root mean squared error (RMSE = 8827); and second, predicting a probable end date found to be around the end of December 2020 with a projected number of 378,299 at the end of the outbreak. The obtained results may help the decision-makers to take suitable decisions related to the pandemic mitigation and containment and provide clear understanding of the virus dynamics in Saudi Arabia.

## 1. Introduction

In early 2019, the COVID-19 disease has started spreading from Wuhan city, Hubei province in China. Few days later, it quickly spread across the world infecting 39,170,503 persons and causing the death of 1,102,926 as of October 16, 2020. Although 29,378,739 individuals have recovered/discharged, 8,688,838 are reported as still active cases [1]. Several actions have been implemented by different countries ranging from total lockdown to social distancing measures in order to contain the virus and limit its spread [2]. Moreover, economic activities and health systems around the globe have been extremely affected which made the world in front of an unprecedented difficult situation.

Large attention is being paid by scientists from different backgrounds since they are hardly working on COVID-19 various aspects including the virus dynamics modeling. Several researches have been published aiming to predict the number of infected cases, the number of deaths and more particularly a probable end date in different countries and sometimes in different cities (provinces) inside the same country. As examples, we can cite references [3] for Saudi Arabia, [4] for Kuwait and [5] for India. The reported results have shown varying degrees of accuracy and reliability due to several causes including the quality and quantity of available information about the virus [6]. Suspected-Infected-Recovered (SIR) model as well as its variants have been used to predict the virus dynamics [2],[4],[7]. Although these models are continuous-time and the data of reported cases are available in discrete-time frequency (daily), several studies have considered SIR based on discrete-time frameworks [8]–[10]. The effect of containment and control measures has been also considered by using appropriate values of the model parameters. Moreover, time-series models such as autoregressive moving average (ARIMA) [11]–[12] and its variants in addition to different artificial intelligence (AI) based models are facing luck of sufficient information both quantitatively and qualitatively [5]–[6],[10],[12].

The logistic growth model and many of its variants have been utilized for predicting COVID-19 future in different countries. For example, the study in [3] have developed simultaneously SIR and logistic growth models for the case study of Saudi Arabia using data between March 2^nd^ and May 15^th^, 2020. Unfortunately, by checking back their forecasted cumulative infected numbers and projected end date, it has been found that their projections were inaccurate since they expected a number ranging between 69,000 and 79,000 at the end of June 2020 as probable end date (the real number was 190,823 in June 30, 2020) [13]. The case study of Kuwait using logistic regression models has been examined in [4]. The predicted and real total infected numbers are checked and found to be different at the projected end date (predicted: 4,100, real: 17,700) [14]. Five-parameter logistic growth model has been used for reconstructing COVID-19 data in USA [15]. Results showed a good fit and a relatively accurate estimation of new infected cases as for April 4^th^, 2020 but unfortunately the developed model has failed in predicting the end date since the virus continues its increase till today (October 16, 2020). The case study of India has been also investigated using logistic growth model in [16]. The developed model has been found to over-estimate the total number of cumulative infected cases by May 22, 2020 (predicted: 1 million, reported: 124,000). The drawback of the logistic growth model is that it is suitable only for modeling early stages of epidemics [17]. Combined models have been also considered for predicting COVID-19 [18]–[20]. A combination of logistic model with machine learning [18], ANFIS with virus optimization algorithm [19] and Gaussian mixture model are examples of such frameworks reported to be efficient. As a conclusion, it is clearly observed that most of the developed models succeeded in fitting the historical data but faced difficulties in predicting the number of infected cases in the coming future.

Until now, based on the above discussed references and to the best of the authors' knowledge, few researches have used phenomenological models for COVID-19 forecasting. Moreover, few studies that considered using Richards model combined with a global optimization swarm intelligence technique (the Particle Swarm Optimization (PSO)) for predicting COVID-19 dynamics are available. For this aim, this study will focus on implementing phenomenological models, namely, generalized growth model (GGM), generalized logistic model (GLM), classical logistic growing model (CLGM) and generalized Richards model (GRM) to predict the dynamics of COVID-19 dynamics in Saudi Arabia. SIR model will be also implemented on the same dataset for comparison purpose. The main motivations behind this choice are summarized as follows: (i) this class of models is known to be robust, (ii) they include few parameters to be calibrated optimally and (iii) COVID-19 (like any other epidemic) should pass through an exponential growing phase at the beginning, should reach a peak and should exhibit a flat and stable curve which is well-captured by both CLGM and GRM. In this study, the identification of the models' parameters is defined as a minimization problem. The quadratic error between the cumulative infected cases and the reported infected cases is minimized by respect to the model parameters. The identification problem is then solved using the global PSO technique. The uniqueness and the stability of the developed models will also be investigated.

The remaining of this paper is organized as follows. Section 2 presents the models investigated in this study as well as details about the computational methodology used to identify the models' parameters. In Section 3, the results of the conducted experiments using Saudi Arabia case study are provided. The obtained results will be discussed in Section 4. Finally, conclusion and recommendations are provided in Section 5.

## 2. Materials and methods

In this section, the four phenomenological models as well as the SIR compartmental model used to forecast the dynamics of COVID-19 in short-term horizon are presented and discussed in detail [21]–[23],[33],[34].

### 2.1. Generalized growth model (GGM)

The generalized growth model is described in Eq 1 below:

$$\frac{dC(t)}{dt}=rC{\left(t\right)}^{p}$$ (1)

This model is usually used in early stages of an epidemic spread. It relies on two parameters; namely the intrinsic growth rate, *r* and the scaling growth rate, *p*. According to the value of *p*, three growth profiles can occur: a straight linear behavior (*p* = 0), an exponential growth (*p* = 1) and a sub-exponential growth (*p* < 1). In a model identification framework, the parameter *p* search space is set to be the interval [0–1]. *C*(*t*) denotes the cumulative number of infected cases and $\frac{dC(t)}{dt}$ is its derivative with respect to the time, t.

### 2.2. Classical logistic growth model (CLGM)

The classical logistic growth model is described in Eq 2.

$$\frac{dC(t)}{dt}=rC\left(t\right){\left(1-\frac{C\left(t\right)}{K}\right)}^{\;}$$ (2)

The model parameters are the same as in the GGM. This model is expected to capture both the early stage exponential curve and a steady state (flat curve) reached at the end of the epidemic. During the late stages of the virus, the number of new cases becomes small and the number of cumulative cases becomes constant equal to the final capacity, K.

### 2.3. Generalized logistic model (GLM)

The generalized logistic model is described in Eq 3 below:

$$\frac{dC(t)}{dt}=rC{\left(t\right)}^{p}\left(1-\frac{C\left(t\right)}{K}\right)$$ (3)

This model is similar to the CLGM except of the inclusion of a scaling growth rate, *p*.

### 2.4. Generalized Richards model (GRM)

The generalized Richards model is described in Eq 4 below:

$$\frac{dC(t)}{dt}=rC{\left(t\right)}^{p}{\left(1-{\left(\frac{C\left(t\right)}{K}\right)}^{\alpha }\right)}^{\;}$$ (4)

This model includes all the features covered by the previous three models and it includes an exponent factor *α* used to capture the deviation of the symmetric S-shaped dynamics of the simple logistic model. It is known to be pertinent since it can capture the epidemic curve in all its phases.

### 2.5. Suspected-Infected-Recovered (SIR) model

In this paper, the SIR model is adopted since it has been reported to be simpler than other complicated models such as SIER [8],[35]–[37]. It will be used for comparison with the developed four logistic models. SIR model includes a set of differential equations (in continuous-time) describing the relationships between subsets of a population including Suspected (S), Infected (I) and Recovered (R) [9]–[10]. In SIR model, the dynamics of the COVID-19 are described as follows Eq 5–Eq 7:

$$\frac{dS(t)}{dt}=-KS\left(t\right)I\left(t\right)$$ (5)

$$\frac{dI\left(t\right)}{dt}=KS\left(t\right)I\left(t\right)-\frac{1}{\beta }I\left(t\right)$$ (6)

$$\frac{dR(t)}{dt}=\;\frac{1}{\beta }I\left(t\right)$$ (7)

where *K* is the contact rate expressing the probability of being infected and *β* is the characteristic duration of the disease.

Since the COVID-19 pandemic data are available in a discrete-time level (daily), finding SIR model solutions should be based on efficient algorithms operating on discrete-time [9]. For this purpose, the first-order Euler method is used for transforming the SIR continuous-time model (Eq 5–Eq 7) into a discrete-time form as follows (Eq 8–Eq 10) (for this purpose, dS(t) = S(t+dt) − S(t) and dt = 1 day):

S(t+1)=S(t)−KS(t)I(t) (8)

$$I\left(t+1\right)=I\left(t\right)+KS\left(t\right)I\left(t\right)-\frac{1}{\beta }I\left(t\right)$$ (9)

$$R\left(t+1\right)=R\left(t\right)+\frac{1}{\beta }I\left(t\right)$$ (10)

where the subscript *t* indicates the day number.

### 2.6. Computational methodology

PSO has been used in the literature for modeling the COVID-19 dynamics. For example, in [24], PSO has been used among other metaheuristic algorithms to tune optimally the parameters of an adaptive neuro-fuzzy inference system (ANFIS) used for predicting the number of infected cases in upcoming days in China. PSO has provided good results in terms of coefficient of determination (R^2^ = 0.9492) and mean absolute percentage error (MAPE = 5.12%). The PSO technique has been also used successfully in calibrating SEIR model for the case study of Hubei, China [25]. In addition to a good data fitting, the PSO algorithm helped in detecting several nonlinear aspects including chaos. The identified model parameters have been used later to establish efficient control strategies. Robust machine learning approach based on PSO has been investigated by [26] to predict the number of infected individuals in Italy. Seven-parameters SEIR model has been found to provide accuracies in the number of susceptible cases ranging from 10% of the population to 40% respectively in Lombardy and Valle d'Aosta.

In this study, the identification of the five proposed models' parameters is performed using a swarm intelligence population-based algorithm, the particle swarm optimization (PSO) [27]. PSO is an emerging optimization technique inspired from animals' social behavior such as bird flocking or fish schooling. It is known to be easy to implement and simple in its intuitive principle [28]. In the PSO principle, the swarm is composed of S particles coding each one a candidate solution for the epidemic model parameters' identification problem. During the optimization procedure, these particles fly across the D-dimensional search-space looking for the optimal position leading to the minimum value of an objective function. The objective function in this study is the nonlinear least-square error between the reported cumulative infected cases and the number of cases calculated using the D parameters of the model associated to each particle. After being initialized randomly at the beginning of the optimization process, the particle move is based on three components: following its current direction, returning back to its best personal position so far visited and moving toward the position reached by the best particle in the swarm. Let i be the index of the i^th^ particle and j be the j^th^ dimension of the model parameters vector. The particle position is defined as:

$$X^{i}=X_{1}^{i}\;,\;X_{2}^{i}\ldots \ldots X_{j}^{i}\ldots \ldots X_{D}^{i}\text{ I}=\text{1},\text{2},\ldots ,\text{S}$$ (11)

To the i^th^ particle are associated three vectors having the same size and the same parameters orders as in X^i^. *P^i^*, *G^i^* and *V^i^* denote respectively the best position so far visited, the position of the best particle among the swarm and the current velocity vector. During the optimization process (with k denotes the iteration index), each particle inside the swarm evaluates its current objective function and updates its position following the set of Eq 12–Eq 14:

$$V_{k+1}^{i}=w_{k}V_{k}^{i}+c_{1}r_{1}\left(P^{i}-X^{i}\right)+c_{2}r_{2}\;\left(G^{i}-X^{i}\right)$$ (12)

$$X_{k+1}^{i}=X_{k}^{i}+V_{k+1}^{i}$$ (13)

$$w_{k}=w_{max}-\frac{w_{max}-w_{min}}{k_{max}}\times k$$ (14)

The inertia weight coefficient is included to create a lance between global search ability at the beginning (high value of the inertia weight) and local search ability at the end of the of optimization procedure. The inertia weight decreases linearly across the iteration index. The computations are stopped when a maximum value of the iteration index will be reached. The adopted solution of the identification problem is reported as the position vector of the global best particle at the final iteration. The optimization problem is then defined as follows:

$$\hat{X}=\arg\min\sum_{t=1}^{t=N}{\left(C\left(t,X\right)-C\left(t\right)\right)}^{2}$$ (15)

$$X_{jl}\leq X_{j}\leq X_{ju}$$ (16)

X_jl_ and X_ju_ denote respectively the lower and upper limits of the j^th^ dimension in X.

For the SIR model, the criterion to be minimized is given below (Eq 17):

$$J\left(X\right)=\overset{t_{f}}{\underset{t_{0}}{\sum }}{\left(S\left(t\right)-\hat{S}\left(t\right)\right)}^{2}+{\left(I\left(t\right)-\hat{I}\left(t\right)\right)}^{2}+{\left(R\left(t\right)-\hat{R}\left(t\right)\right)}^{2}$$ (17)

*t*_0_ and *t_f_* are the first day and the last day of the COVID-19 collected data, respectively. $\hat{S}\left(t\right),\hat{I}\left(t\right)\;and\;\hat{R}\left(t\right)$ are the estimates of S, I and R at the t^th^ day.

## 3. Results

In this section, the results of using the particle swarm optimization (PSO) algorithm described in the previous section for calibrating four phenomenological epidemic models and SIR compartmental model using data of COVID-19 cumulative cases in Saudi Arabia covering the period from March 2^nd^ to October 10^th^, 2020 are provided. The PSO setting parameters are presented in Table 1.

**Table 1.** Parameter settings of the PSO algorithm.

Parameter	Meaning	Value
S	Number of particles	50
***k_max_***	Maximum iteration number used as stopping criterion	200
***w_max_***	Maximum and initial value of the inertia weight	0.9
***w*_min_**	Minimum and final value of the inertia weight	0.4
***c*_1_*and c*_2_**	Cognitive and social factors	0.75
***r*_1_*and r*_2_**	Random numbers	Within [0–1]

The dataset used to calibrate the five previously described models is collected from the Saudi Ministry of Health website covering the period between March 2^nd^ and October 10^th^, 2020. This dataset includes 223 daily observations which have been divided into two subsets: 190 observations used for training the models and the remaining 33 observations used for the models' validation. Testing dataset is composed of the five days immediately following October 10, 2020. This dataset is not included in the training and validation phases. When examining the Saudi Arabia's COVID-19 curves, it is easy to detect that an exponential growth for the cumulative infected cases occurs at the beginning of the pandemic and that around September 20, 2020, the curve starts becoming flat. The new daily reported cases' curve exhibits a clear fluctuating and variable behavior presenting four peaks (May 17, June 17, June 30 and July 6). This fluctuation is correlated with numerous measures implemented by the Saudi authorities to mitigate the virus spread while considering economic and social issues.

To compare the four models' prediction results, three statistical performance indicators, namely, the coefficient of determination (R^2^), the mean absolute percentage error (MAPE), the root mean squared error (RMSE) [27] and the Kolmogorov-Smirnov (K-S) test [29] are used. K-S is a two-sample test used to compare the cumulative distribution probabilities of the reported number of infected individuals and the same number forecasted by one of the investigated models. In Table 2 below, H = 0 indicates that “*Do not reject the null hypothesis at the 5% significance level*” and H = 1 indicates that “*Reject the null hypothesis at the 5% significance level*”.

In this paper, the results of the best run of each model are reported since PSO is a stochastic optimization technique which need to be run many times. Table 2 shows the optimal parameters for each model as well as the values of the performance indicators including the K-S test result.

**Table 2.** Results of the best run of each prediction model.

Model	*r*	*p*	*K*	*α*	Performance indicators				
Search limits	[0–3]	[0–1]	[370,000–500,000]	[0–2]	MAPE (%)	R^2^	RMSE (case)	K-S test	Rank
GGM	1.7628	0.6247	-	-	14.0749	0.8140	72,086	H = 1	5
GLM	1.2986	0.6797	453,459	-	8.8694	0.9615	26,126	H = 0	3
CLGM	0.1262	-	375,082	-	20.6152	0.8694	63,882	H = 1	4
GRM	1.3595	0.6911	378,299	0.9211	3.2889	0.9953	8,827	H = 0	1
SIR	*K* = 2.0593×10^−9^(day^−1^)		*β* = 13.5663 day		4.9898	0.9926	10,700	H = 0	2

**Figure 1.**
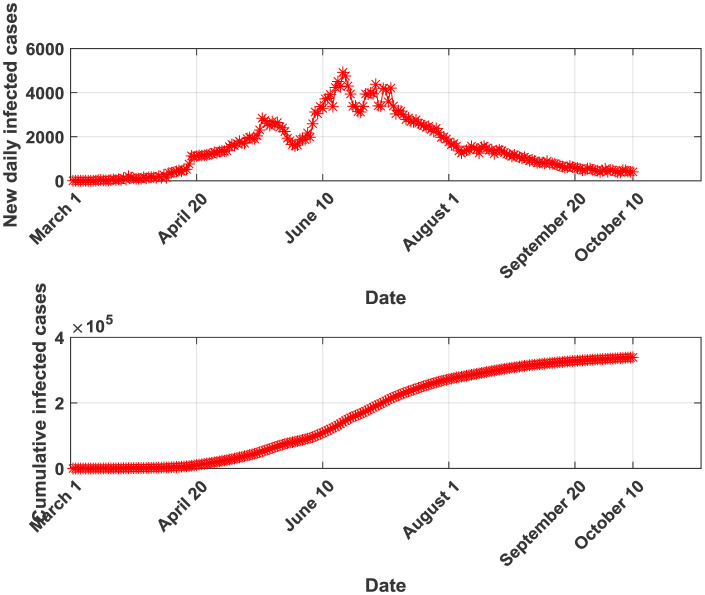
New daily and cumulative infected cases for Saudi Arabia between March 2 and October 10, 2020.

**Figure 2.**
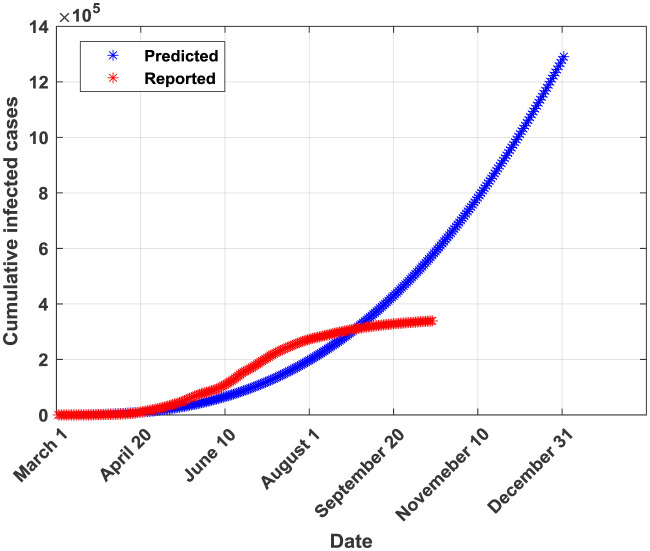
Cumulative infected cases plot for GGM.

**Figure 3.**
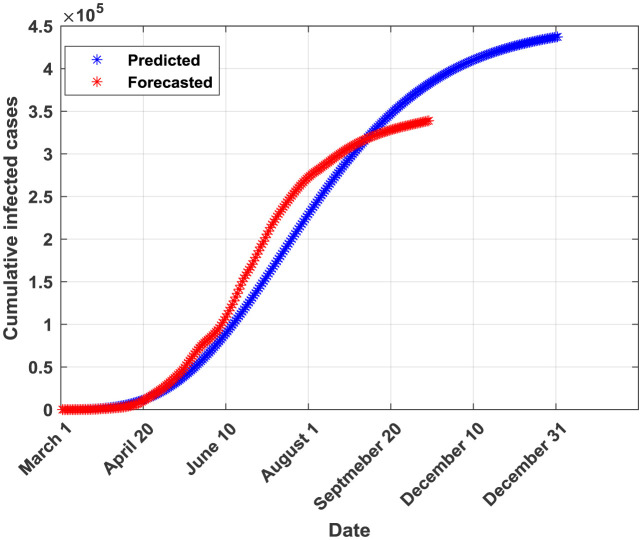
Cumulative infected cases plot for GLM.

**Figure 4.**
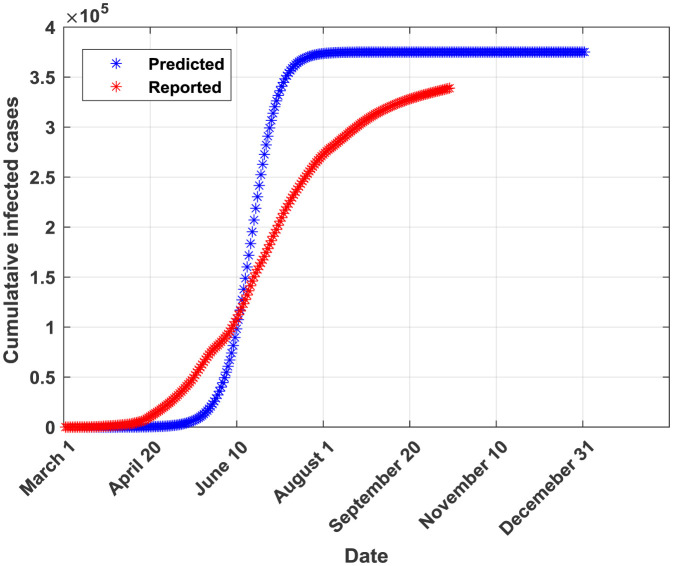
Cumulative infected cases plot for CLGM.

**Figure 5.**
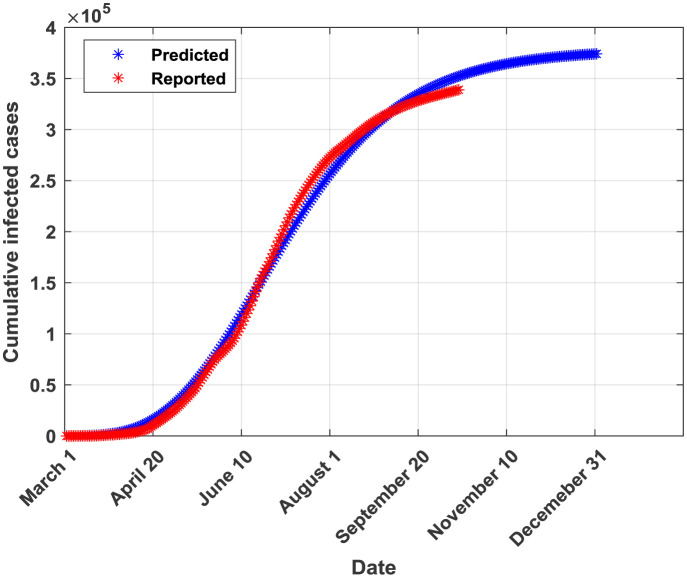
Cumulative infected cases plot for GRM.

The plots of the reported and predicted cumulative infected cases are provided in Figures 2–5.

## 4. Discussion

### 4.1. Comparison between the five developed models

From Table 2, it can be seen that the generalized Richards model (GRM) outperforms all the other four models in terms of all performance indicators. Moreover, this model allows predicting a probable end date (found to be around the end of 2020). The SIR model is ranked in the second place after the GRM in terms of performance metrics. Both GRM and SIR models were valid according to the Kolmogorov-Smirnov K-S test indicating that they provide forecasted and real (reported) infections derived from similar empirical distribution functions [29]. Both models provide also almost similar values of the coefficient of determination R^2^ (0.9953 for GRM and 0.9926 for SIR). This result can be attributed to the fact that logistic models are rigorously derived from the simple epidemiological SIR model as reported in [9],[30]. The third-ranked model is found to be the GLM. In fact, it provided a probable end date near from the one provided by the best model (the GRM) but the number of infected cases at the end of the pandemic is higher than the one provided by the GRM. The GLM model is found to be valid according to the K-S test. The fourth model is CLGM. In fact, it yielded a final capacity of the virus almost near of the one issued from the best model (GRM) but an end date around the end of August, 2020 which is not true according to the current COVID-19 statistics in Saudi Arabia. Moreover, the K-S test was non valid for the CLGM model. It should be noted that, according to the curve in Figure 4, the CLGM has a chance to meet the end of the virus capacity since the real curve is still under the curve provided by the CLGM. The last model is the GGM. This model is found to be good in fitting data for the early stage of the epidemic but it becomes inaccurate in the coming stages [31]. Conceptually, the GGM model cannot capture the probable end of the outbreak since its curve will continue growing with time. In Table 3, the actual and forecasted (by the GRM) cumulative number of infected cases are provided for the validation phase. The differences are relatively small which confirms the good fit performance of the GRM when applied to a dataset not used during the training phase.

**Table 3.** Reported and predicted cumulative infected cases for the validation phase (day#1 is September 8,2020): results of the GRM.

Day#	Reported	Predicted	Day# number	Reported	Predicted	Day# number	Reported	Predicted
1	322,237	325,176	12	329,271	336,570	23	334,605	345,683
2	323,012	326,317	13	329,754	337,487	24	335,097	346,412
3	323,720	327,436	14	330,246	338,385	25	335,578	347,125
4	324,407	328,533	15	330,798	339,265	26	335,997	347,824
5	325,050	329,609	16	331,359	340,127	27	336,387	348,508
6	325,651	330,664	17	331,857	340,971	28	336,766	349,177
7	326,258	331,699	18	332,329	341,798	29	337,243	349,832
8	326,930	332,713	19	332,790	342,608	30	337,711	350,474
9	327,551	333,706	20	333,193	343,401	31	338,132	351,101
10	328,144	334,680	21	333,648	344,177	32	338,539	351,715
11	328,720	335,635	22	334,187	344,938	33	338,944	352,316

**Table 4.** Reported and predicted cumulative infected cases for the testing phase for a period of five days: results of the GRM.

Date	Reported	Predicted by GRM	Relative error
October 11, 2020	339,267	352,904	3.9984%
October 12, 2020	339,615	353,480	4.0652%
October 13, 2020	340,089	354,043	4.0913%
October 14, 2020	340,590	354,593	4.1057%
October 15, 2020	341,062	355,132	4.1253%

In Table 4, the number of cumulative infected cases provided by the GRM as well as real reported numbers are compared. A relative error around 4% is found to characterize the model. Note here that the considered five days were not included in the model training and validation phases.

### 4.2. Analysis of the generalized Richards model (GRM) forecasts

The generalized Richards model (GRM) is found to outperform all the other four models developed in this paper. Therefore, its main features including the advantages, the limitations and the robustness will be discussed in detail. In forecasting exercises, the data available at the hand of the designer is usually divided into 80% for training the model and 20% for validating it. However, when investigating our models including the GRM, we have tried other choices for the two subsets and we found that this didn't affect a lot the overall forecasting performances more particularly for the case of the GRM. One of the main drawbacks of the GRM is that it can't predict the fatality curve like in other researches such as [38].

Possible sources of errors in final estimates of the cumulative number of infections have been included in the models' parameters through the particle swarm optimization (PSO) algorithm. In fact, during the parameters' identification process, the vector of parameters is perturbed using the r_1_ and r_2_ random numbers (see Table 1: parameter settings of the PSO and Eq 12: particle move). A stretched logistic equation for COVID-19 spreading in Italy has been proposed in [30]. This model is expected to take into account the time-dependency of the growth rate. By comparing the curve provided in Figure 6 of [30] and the curve provided by our GRM model (Figure 5 of this paper), we can conclude that our model is behaving similarly to a stretched logistic model with the difference that in Saudi Arabia, COVID-19 pandemic didn't reach the flat curve, indicating the end of the virus current wave, yet.

In order to highlight the effect of the changing situation of transmissibility, several scenarios related to intermediate date ranges have been investigated for the GRM as representative of the studied four logistic models. The GRM has been calibrated respectively using the first 100, 150, 200 and 223 days used for the model training. The results of different scenarios are summarized in Table 5.

**Table 5.** Results of the GRM in different time scenarios.

Parameters' search limits	r	p	K	α	Performance indicators				
Time range scenarios	[0–3]	[0–1]	[370,000–500,000]	[0–2]	MAPE (%)	R^2^	RMSE (case)	K-S test	Rank
100	1.7003	0.6921	454,842	0.3088	2.9012	0.9324	7,078	H = 0	4
150	1.2365	0.6825	441,454	1.5705	5.0476	0.9604	16,308	H = 0	3
200	0.7413	0.7821	373,468	0.5965	5.9539	0.9842	16,383	H = 0	2
223	1.3595	0.6911	378,299	0.9211	3.2889	0.9953	8,827	H = 0	1

As depicted in Table 5, the four runs' parameters are extremely different which reflects relatively high variability of the growth dynamics of the epidemic. The situation of transmissibility is found to be changing. This fact is also clear in Figure 1 where it is shown that the curve of new infections presented four local peaks. Obviously, the coefficient of determination R^2^ increases when the length of the sample used for training the GRM increases. This confirms also that using more data for training may allow detecting different dynamics of the COVID-19 pandemic and therefore being able to explain the effect of the virus containment measures [39] and the efficiency of the social behavior including isolation like in the case of Brazil [40].

• The GRM model has been trained using other parameter fitting techniques (genetic algorithm (ga) and Levenberg-Marquardt (LM)) in order to show the superiority of the particle swarm optimization technique. The PSO is found to be more efficient at least in three points: (1) The PSO has global search ability since the particles are initialized randomly over the whole search space at the beginning of the identification process; (2) The particles are then driven towards the “sub-optimal” solution. Following the inertia weight coefficient which favors the “exploration” at the beginning and favors the “exploitation” at the end of the identification process; and (3) The PSO has been found to operate in complex search-spaces that should be non-convex. However, both LM and “ga” have been trapped into local minima since they are sensitive to the initialization.

• The PSO technique used in this paper to calibrate all the developed models is known to be stochastic since it initializes the particles randomly within the search-space limits and it includes two numbers (r_1_ and r_2_) which are generated randomly inside the interval [0–1] at each iteration. The two random numbers multiply respectively the local search component and the global search component (see Eq 12). The particle move includes through the use of r_1_ and r_2_ some kind of perturbations reflecting thus the robustness of the derived solutions. Due to this random aspect, the algorithm has been run several times and the results of the best run are adopted. Thus, the PSO is found to yield “sub-optimal” solutions that are not unique. A trade-off between optimality and feasibility is ensured.

• In order to compare our approaches to other approaches applied to the case study of Saudi Arabia, three references studying the prediction of the COVID-19 number of infected cases are selected. The results of comparison are included in Table 6 below.

It can be concluded from this comparison that the results are extremely variable since the forecasting performance metrics depend on the length and quality of the available data, the virus stage and transmissibility variation, the measures taken by the local authorities and on the used method. Forecasting is then a case-sensitive exercise.

**Table 6.** Comparison of the prediction of COVID-19 infected cases in Saudi Arabia.

Ref.	Used dataset	Method/Technique	Performance metrics	Comments
[11]	Daily from March 2, 2020 to April 20, 2020	Times-series ARIMA model and its variants for predicting the next four weeks infections	• R^2^: ranges from 0.46 to 0.99 • MAPE: ranges from 2.6% to 32.80%	Although the performance metrics are reported to be good, checking back the forecasted values showed the model to overestimate the number of cumulative infected case in the four weeks after the end of the dataset as well as an inaccurate end date of the outbreak
[3]	Daily from March 2, 2020 to May 15, 2020	Logistic growth and SIR models for predicting more than one month of daily infected cases	• Growth model end date around the end of June 2020 with about 70,000 of total cases • SIR model end date was forecasted to be around the end of June and a cumulative number of 80,000	Both models are found to be inaccurate since the virus continues spreading as of October 18, 2020
[32]	Daily from March 2, 2020 to May 14, 2020	Using a modified SIR model including parameters related to temperature, humidity, population density and the intensity of control measures taken by local governments.	Daily new cases found to range from 1,800 to 2,500 between May 15, 2020 and May 26, 2020	Forecasts where relatively accurate when compared to the reported cases
GRM (this work)	Daily from March 2, 2020 to October 10, 2020	Using the generalized Richards Model (GRM) for predicting cumulative infected cases	• R^2^: 0.9953 • MAPE: 3.2889% • Predicted end date: end of 2020 • Predicted cumulative number at the end of the outbreak: 378,299

### 5. Conclusion

In this paper, a modeling procedure based on phenomenological and compartmental models combined with the particle swarm optimization (PSO) technique is carried out using reported cases from Saudi Arabia for the period starting on March 2^nd^ and ending on October 10, 2020. The cumulative infected cases and a probable end date of the COVID-19 pandemic are predicted using five models including the four-parameter generalized Richards model (GRM). This model has provided good fit of data and a probable projected end date around the end of 2020 with a total number of infected cases around 379 thousand. According to the study results, it has been shown that the COVID-19 outbreak is approaching its end. The Saudi experience can be considered as successful in containing the first wave until now and more efforts in enforcing social distancing should contribute to mitigate the virus.
